# Evaluation and implementation of the 4 hour ics core standard: 2 year audit across the greater manchester critical care network

**DOI:** 10.1186/2197-425X-3-S1-A762

**Published:** 2015-10-01

**Authors:** AJ Parker, JH Littler, JM Eddleston

**Affiliations:** Greater Manchester Critical Care Network, Manchester, United Kingdom

## Introduction

The Intensive Care Society (ICS) of the UK introduced *'Core Standards for Intensive Care Units'* in 2013^1^. It outlined a standard that a patient should be admitted to a Critical Care Unit within 4 hours of making the initial decision. This necessitates accurate recording of key decision times in patient notes or other hospital systems for calculation of an accurate time interval to admission.

Currently there is no specifically agreed format or data submission system to capture this.

## Method

We established an annual month long audit across Greater Manchester Critical Care Network (GMCCN) to examine compliance with the ICS standard for unplanned access to Critical Care.

We undertook prospective analysis of consecutive unplanned admissions to 9 Intensive Care Units (ICU), recording the time of the decision to admit to Critical Care and actual admission time. After year 1, results and barriers to implementation were fed back to individual sites to allow them to try and improve performance and data capture. The audit was repeated 1 year later.

## Results

**Figure 1:**
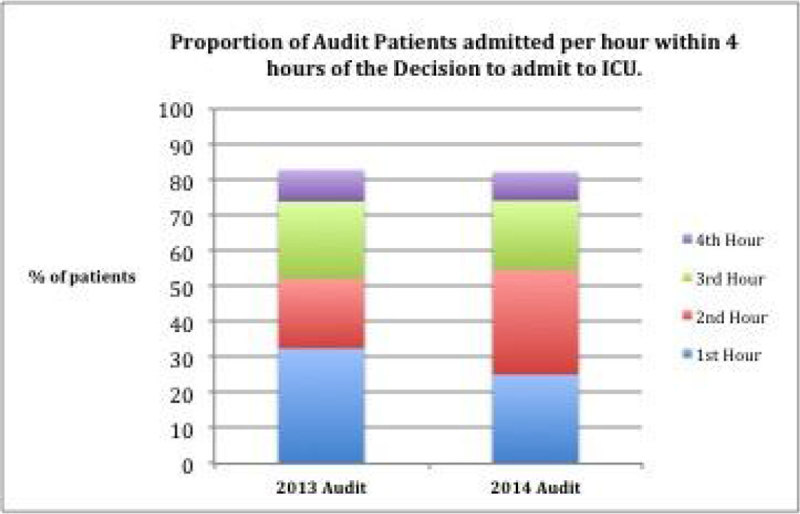
**Compliance with 4 hour admission standard.**

Table 1 shows audit data across 2 years. Figure 1 shows the proportion of patients admitted within the first four hours from the decision to admit to critical care. Overall 82.8% were admitted within 4 hours in 2013 vs. 82.3% in 2014.

**Table 1:** Numbers in audit 2013-2014

Year	ICU Patients in Audit	Patients for whom delay was calculable	Proportion of audit group with a calculable time from decision to admission (%)
**2013**	204	157	76.9%
**2014**	217	175	80.6%

Further analysis of 2013 data showed only 82% of total unplanned admissions across the network were captured in the audit.

## Conclusions

Data capture has improved over two years within GMCCN but overall performance is unchanged. Continued audit is required to measure compliance and drive improvement.

There are significant barriers to data capture. Encouraging clinicians to document the time of their decision to admit a patient remains a challenge within paper based records. Extracting information for audit requires additional work that may not capture all patients and limits audit frequency.

In many hospitals, electronic data management systems do not record the decision to admit to Critical Care in the same way that admission time is recorded. Integrating this function into electronic patient/ bed management systems would improve data and could be utilised in real time to drive performance of care providers.

Agreement as to the precise time of the decision to admit varies between clinicians. Immediate stabilization may take considerable time. Requesting an admission before or after appropriate clinical delays e.g. trips to radiology impacts on timing. This needs to be considered, particularly if the ICS standard were to be linked to penalties for non-compliance.

High quality care remains the aim of standards rather than achievement of the target. Careful implementation should seek to ensure that the goal remains that care is delivered in the right place at the right time by the right team.
